# Updates on Anticancer Therapy-Mediated Vascular Toxicity and New Horizons in Therapeutic Strategies

**DOI:** 10.3389/fcvm.2021.694711

**Published:** 2021-07-27

**Authors:** Po-Yen Hsu, Aynura Mammadova, Nadia Benkirane-Jessel, Laurent Désaubry, Canan G. Nebigil

**Affiliations:** INSERM UMR 1260, Regenerative Nanomedicine, University of Strasbourg, FMTS (Fédération de Médecine Translationnelle de l'Université de Strasbourg), Strasbourg, France

**Keywords:** vascular toxicity, anti-cancer drugs, cardiotoxicity, hypertension, thrombosis

## Abstract

Vascular toxicity is a frequent adverse effect of current anticancer chemotherapies and often results from endothelial dysfunction. Vascular endothelial growth factor inhibitors (VEGFi), anthracyclines, plant alkaloids, alkylating agents, antimetabolites, and radiation therapy evoke vascular toxicity. These anticancer treatments not only affect tumor vascularization in a beneficial manner, they also damage ECs in the heart. Cardiac ECs have a vital role in cardiovascular functions including hemostasis, inflammatory and coagulation responses, vasculogenesis, and angiogenesis. EC damage can be resulted from capturing angiogenic factors, inhibiting EC proliferation, survival and signal transduction, or altering vascular tone. EC dysfunction accounts for the pathogenesis of myocardial infarction, atherothrombosis, microangiopathies, and hypertension. In this review, we provide a comprehensive overview of the effects of chemotherapeutic agents on vascular toxicity leading to hypertension, microvascular rarefaction thrombosis and atherosclerosis, and affecting drug delivery. We also describe the potential therapeutic approaches such as vascular endothelial growth factor (VEGF)-B and prokineticin receptor-1 agonists to maintain endothelial function during or following treatments with chemotherapeutic agents, without affecting anti-tumor effectiveness.

## Introduction

Anticancer chemotherapies target the vasculature of both tumor and unfortunately other organs. Additionally, mechanism-independent (“off-target”) effects of chemotherapies also account for the development of the vascular toxicity. Vascular toxicity occurs during acute chemotherapeutic regimen, and after once treatments have ceased, persists into survival. The susceptibility to develop vascular complications following chemotherapeutics also relates to many factors such as cardiovascular risk and pre-existing vascular diseases, as well as genetic predispositions.

Chemotherapeutics-mediated vascular toxicity often results from loss of endothelial cell (EC) functions (1). ECs sense hemodynamic changes, and accordingly respond to stimuli by the release of vasoactive substances like vasorelaxants such as nitric oxide, (NO), prostacyclin, (PGI_2_), vasoconstrictors such as endothelin-1, (ET-1), anti-thrombotic (plasminogen activators), and angiogenic factors such as vascular endothelial growth factor (VEGF) (2) and prokineticins (3). Disturbance of NO/ET-1 balance is a characteristic of endothelial dysfunction and play an important role in the progression of vascular diseases.

**Figure 1:**
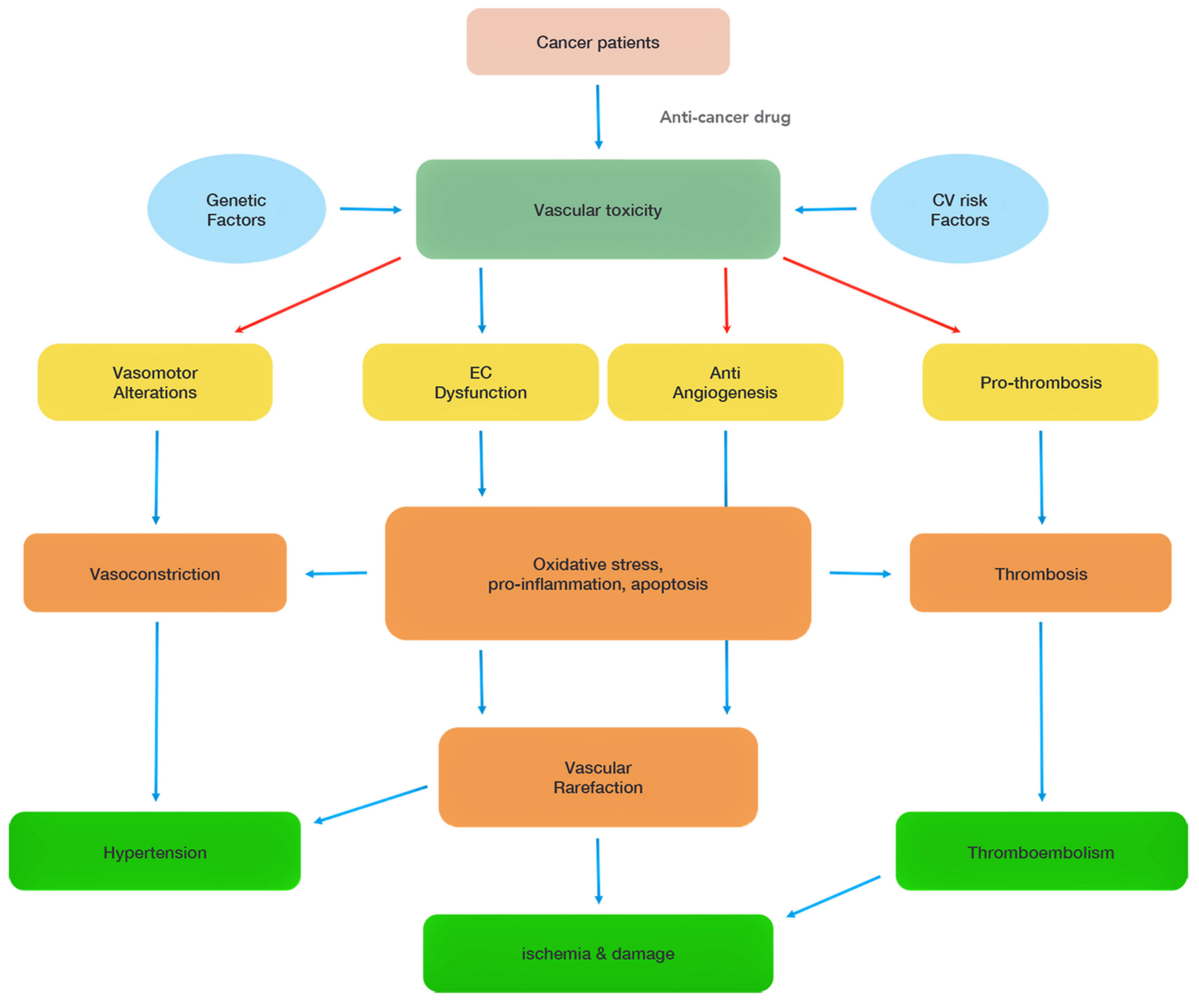
Summary of chemotherapy-associated vascular toxicity. Vascular toxicity can be attributed to four main mechanisms (vasomotor alterations, endothelial cell (EC) dysfunction, anti-angiogenesis, pro-thrombosis), which induce hypertension, ischemia and thromboembolism, and damage heart functions. The red arrows highlight direct effects of some of the chemotherapeutics.

Chemotherapeutics-mediated EC dysfunction in the heart is initially asymptomatic. The long-term consequences of cancer treatments can lead to the onset of cardiovascular disorders such as hypertension, coronary artery disease, and heart failure. Indeed, progressive EC damages make ECs more vulnerable to chronic inflammatory stressors and hyperlipidemia insults (4). EC dysfunction further promotes thrombus formation, and inflammation by releasing plasminogen activator inhibitor 1 (PAI1), platelet-activated factor 4 (PF-4), and interleukins (IL-1 and IL-6) to accelerate atherosclerosis formation. Chemotherapeutics can also have direct pro-coagulant, anti-angiogenesis, and vasoconstriction effects (Figure 1).

**Figure 2:**
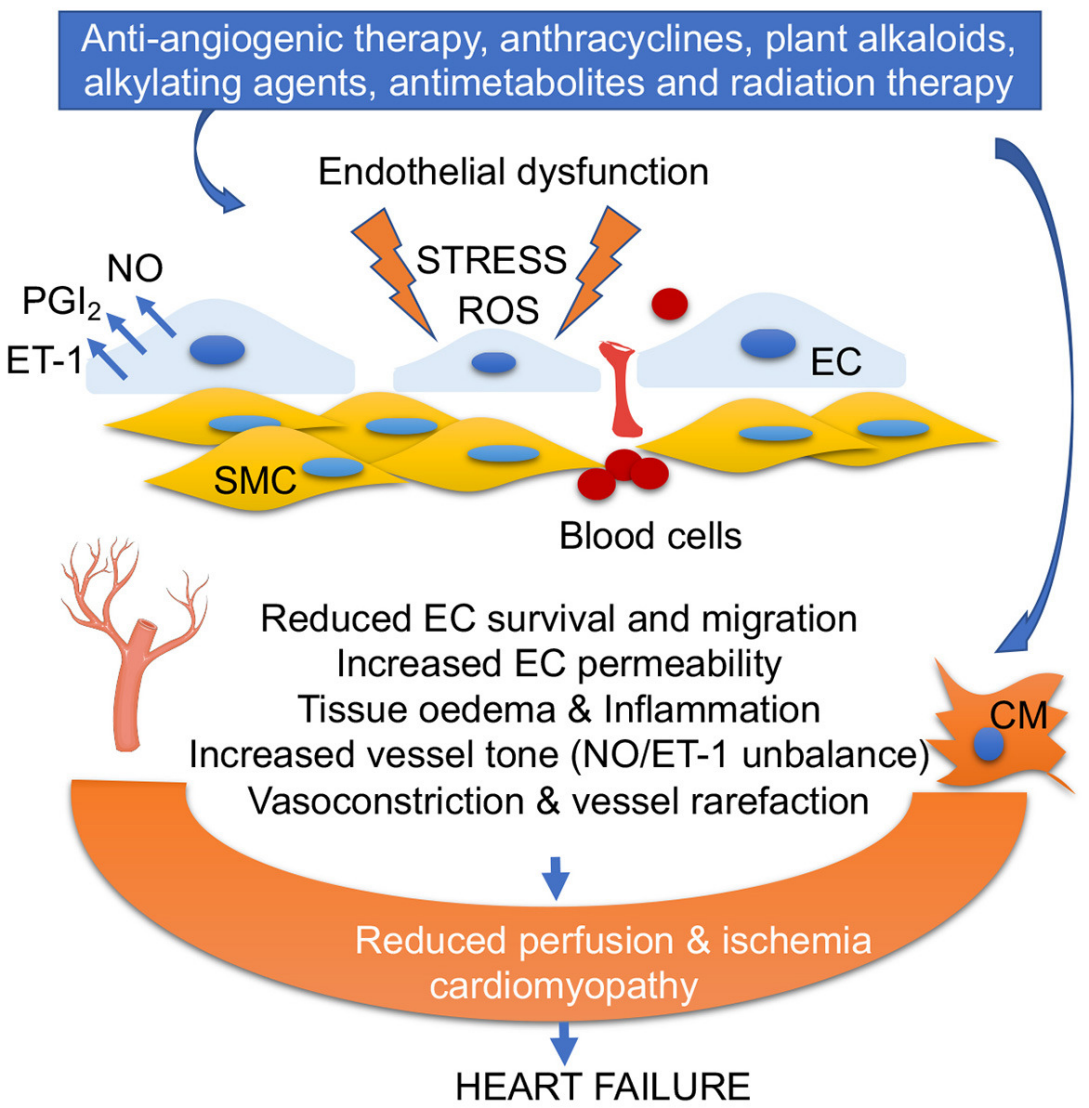
Anticancer drug-mediated endothelial dysfunction. Oxidative stress, ROS accumulation, alteration of PGI_2_/ET-1 ratio, and low NO levels in ECs impair EC survival and migration, increase fenestration and blood cell infiltration into vascular smooth muscles (SMC), trigger inflammation, and promote vasoconstriction. EC dysfunction and vascular rarefaction reduce myocardial perfusion, leading ischemia, and cardiomyopathy. Anticancer drug-mediated cardiomyocyte (CM) damage together with EC dysfunction leads to heart failure.

Vascular damage in the cardiovascular system can be caused not only by anti-angiogenic chemotherapy (inhibitors of vascular endothelial growth factor (VEGFi), but also by anti-tumor antibiotics (bleomycin and anthracyclines) (5, 6). The first line of treatments includes monoclonal antibodies (e.g., bevacizumab), and multiple kinase inhibitors such as sunitinib, a multi-targeted inhibitor, or sorafenib (7). In addition, plant alkaloids (taxanes, vinca alkaloids), alkylating agents (cisplatin, cyclophosphamide), antimetabolites (5-fluorouracil), and radiation therapy also foster vascular damages (8) (Figure 2).

Hereafter, we concentrate on these anti-cancer drugs-mediated vascular damages that evoke cardiovascular diseases and impair drug delivery.

### Anticancer Therapy-Mediated Oxidative Stress and Vascular Injury

Many chemotherapeutics induce accumulation of the reactive oxygen species (ROS) products (9) that disrupt intracellular homeostasis and damage proteins, lipids, and DNA in the vascular cells. ROS such as superoxide radical anions (O_2._^**−**^), lipid radicals (ROO.^**−**^), hydroxyl radicals (HO.^**−**^), and nitric oxide (NO) are formed by all vascular layers, including endothelium, smooth muscle, and adventitia (10). ROS induces VEGF expression in vascular endothelial and smooth muscle cells by upregulating hypoxia-inducible transcription factors (HIF-1). VEGF further stimulates the accumulation of ROS through activation of NADPH oxidase (11). The NO itself has a cardiovascular protective properties (12). However, when NO combines with ROS, it generates peroxynitrite radicals (ONOO^**.−**^) that promote inflammation, apoptosis, necrosis, and ultimately toxicity (13).

A high production of ROS is also a major promoter for the lipid peroxidation of unsaturated fatty acids, leading to apoptosis, autophagy, and ferroptosis (14). Lipid peroxidation followed by the activation of phospholipase A2 initiates the activation of arachidonic acid (AA) pathway. Thus, lipid peroxidation is not only responsible for the generation of prostaglandins, but also for the induction of inflammation and apoptosis in vascular ECs (14). ROS also promotes peroxidation of a mitochondrion-specific inner membrane phospholipid, cardiolipin to activate intrinsic apoptosis (15). Lipid peroxidation products can bind to specific mitochondrial and autophagy-related proteins driving autophagic cell death (16). Elevated intracellular iron concentration elevates ROS levels that cause lipid peroxidation and consequently to ferroptosis-mediated cell death (17).

Reactive nitrogen species (RNS) are formed by the reaction between ROS and NO that damage mitochondrial DNA. Excessive ROS also induces senescence in endothelial, vascular smooth muscle cell (VSMC), and endothelial progenitor cells (18). Indeed, accumulation of ROS and oxidative stress reduces NO bioavailability and consequently results in development of hypertension (19) (Figure 2).

### Anticancer Therapy-Mediated Endothelial Dysfunction and Hypertension

Approximately 25% of cancer patients develop hypertension due to adverse effects of VEGFi, TKI, anthracyclines, alkylating agents, and antimetabolites (20). The pathophysiology of hypertension induced by these agents is not fully elucidated. Several mechanisms have been proposed based on the preclinical and clinical studies, including; (1) increased total peripheral resistance induced by endothelial dysfunction due to predominantly the reduced production of vasodilators (NO and PGI2), the increased production of vasoconstrictors (ET-1) and the reduced nitric oxide bioavailability, (2) increase in vascular tone, (3) vascular rarefaction, (4) and renal thrombotic microangiopathy, leading to proteinuria and hypertension, (5) natriuresis and impaired lymphatic function could also contribute to development of hypertension (21).

#### Inhibitors of VEGF (VEGFi) or Tyrosine Kinase (TKI)

Approximately 80% of patients treated with VEGFi or TKI manifest hypertension (22). VEGF signaling promotes production of NO and the vasodilatory prostanoid prostacyclin (PGI_2_) through activation of phospholipase A2 *via* PLCγ/PKC pathways (23). After VEGF binding, VEGF receptor (VEGFR) activates phosphoinositol-3 kinase (PI3K)/serine-threonine protein kinase B (Akt) survival pathway in ECs. Thus, interruption of the VEGF signaling pathway by anticancer drugs leads to development of hypertension. Similarly, the VEGF trap aflibercept promotes hypertension (24), interrupting VEGF-mediated vasodilatory, and survival signaling (25). VEGFi-induced vascular toxicities can also be due to accumulation of ROS and down-regulation of nuclear factor erythroid 2-related factor 2 (Nrf2) that regulates antioxidant genes (26). Prohypertensive effects of VEGFi can also be promoted by microparticles of injured ECs (27).

TKIs stimulate ROS accumulation and reduce NO levels (28). For example, vatalanib or sunitinib increases ROS accumulation in both VSMCs and ECs by inhibiting NO synthase (NOS) thereby reducing NO levels and decreasing endothelium-dependent vasorelaxation (29). Sunitinib-induced hypertension may not depend on endothelium, but may be due to decreased arterioles diameters. Indeed, it inhibits platelet-derived growth factor receptor (PDGFR) that causes coronary microvascular dysfunction due to loss of pericytes, leading to the mechanical instability of the capillary wall in cardiac and other tissues (30).

#### Anthracyclines

They cause ≈20% increase in carotid artery stiffness in patients. Anthracyclines also led to a 3-fold increase in vascular stiffness with a 10-year follow-up period in adolescent childhood cancer survivors, indicating that alterations in vascular integrity persist years to decades following anthracycline chemotherapy (31). Anthracycline-induced endothelial toxicity and hypertension can be caused by several mechanisms. The first one is an oxidative stress-mediated process (32). Indeed, doxorubicin binds to endothelial (eNOS) and decreases NO levels, leading to the production of superoxide. Reduced concentration of NO shifts endothelium to a pro-coagulant status and impairs vasodilatation (33). Recently, doxorubicin has been shown to stabilize NRF2 in the cytoplasm thereby reducing detoxification pathway in mice heart (6). Doxorubicin also induces mitochondrial DNA damage in an RNS/ROS-independent manner, along with a possible decrease in B-cell lymphoma (Bcl)-2, that leads to apoptosis of the ECs. The EC death further reduces the availability of NO, ET-1, PGI_2_, and neuregulin (NRG)-1 to cardiomyocytes. Indeed, accumulation of ROS and oxidative stress reduces NO bioavailability and consequently results in development of hypertension (34). The second mechanism is apoptosis due to DNA interference (35). Doxorubicin -mediated topoisomerase II-β inhibition and DNA-binding directly induce DNA damage and apoptosis in ECs (36). Doxorubicin also reduces the tight junction protein zona occludens (ZO)-1 in ECs, thereby, increasing microvascular permeability (37). Anthracyclines at the accumulative dosage dysregulate renin-angiotensin-aldosterone (RAA) system (38), that play significant role in the development of hypertension (39).

#### Alkylating Agents

Cyclophosphamide or its metabolites reduce vasoactive substance NO, increase ET-1 and inducible (i) NOS (40). They activate the toll-like receptor 4 (TLR-4) and causes subsequent activation of mitogen-activated protein kinase (MAPK) and c-Jun N-terminal kinases (JNK) (41). Once activated, these signaling pathways increase the expression of tissue necrosis factor alpha (TNFα), cyclooxygenase-2 (cox-2), prostaglandins (PGs), and interleukins (ILs). Cyclophosphamide also decreases fatty acid binding protein (H-FABP) and carnitine palmitoyl transferase-I (CPT-1) levels, resulting in the accumulation of free fatty acids and reduction of ATP production (42). Reduced ATP levels lead to the accumulation of intracellular calcium, which activates transforming growth factor beta (TGF-β) and the production of pro-inflammatory cytokines. Cyclophosphamide is associated with development of interstitial pneumonia and pulmonary fibrosis, leading to vascular sclerosis, and pulmonary hypertension (40).

Patients treated with cisplatin-based chemotherapy also develop persistent hypertension due to endothelial cell activation, damage, and subsequent endothelial dysfunction (43). Cisplatin induces release of inflammatory substances such as IL-1 and IL-6 from ECs to produce hydrogen peroxide that provoke oxidative stress, and mitochondrial DNA lesions, orchestrating cell death (44).

#### Antimetabolites

5-Fluorouracil (5-FU) induces ultrastructural changes in the endothelium of the heart, as well as in various organs by promoting both accumulation of ROS and autophagy process in ECs (45). Its vascular adverse effects include angina with coronary artery spasm and rarely hypertension (46).

In general, anticancer therapies increases blood pressure, therefore, an antihypertensive therapy can be required in case of diastolic blood pressure (DBP) increase >20 mmHg after initiation of anticancer therapy, yet DBP remains within normal limits (47).

### Anticancer Therapy-Mediated Microvascular Rarefaction

Anticancer drugs induce capillary rarefaction that is described as a reduction of the density of arterioles and capillaries. One of the causes of microvascular rarefaction is a decrease of survival rate of microvascular EC. The second mechanism involve endothelial dysfunction that participates to thrombosis, leading to a further reduction in vascular perfusion, and micro vessel destruction (48). The molecular mechanisms of capillary rarefaction associated with the loss of pericytes due to inhibition of platelet derived growth factor (PDGF) receptor (PDGFR), and inhibition of angiogenesis by blocking VEGF signaling pathway.

#### VEGFi and TKIs

Prolonged TKI treatments lead to capillary rarefaction, due to endothelial dysfunction (25). In addition, disruption of both endothelium-dependent and -independent vasodilatation can also promote intense vasoconstriction and microvascular rarefaction. The vascular rarefaction may also be a consequence of VEGFi-associated hypertension (49). Bevacizumab promotes retinal microvascular dysfunction in humans (50). On the other hand, microvascular rarefaction increases peripheral resistance in the microcirculation, thereby, reducing blood flow and further elevating blood pressure.

#### Anthracyclines

A recent preclinical study has shown that chronic treatment with doxorubicin promotes vessel rarefaction in the heart (6). Moreover, a low dose of doxorubicin inhibits EC motility *in vitro* without causing apoptosis. However, whether doxorubicin provoke hypertension in these mice has not been studied.

#### Alkylating Agents

Cyclophosphamide causes extravasation of proteins, toxic metabolites, and erythrocytes, which breaks-down ECs, promotes hemorrhage, blocks the small arteries, and induces displacement of vascular ECs that directly damages the blood vessels and cardiac cells (51). Cyclophosphamide may reduce VEGF levels that is associated with microvascular rarefaction.

Cisplatin inhibits EC proliferation and motility *in vitro* and causes apoptosis (52). Cisplatin also inhibits angiogenesis (53). Thus, both EC dysfunction and anti-angiogenic effects of platinum derivatives promote vascular rarefaction.

### Anticancer Therapy-Mediated Hypercoagulation, Thrombosis, and Atherosclerosis

Cancer patients exhibit an increased risk of arterial and venous thrombotic events. Approximately cancer patients develop the risk of arterial (2–5%) and venous (4–20%) thromboembolism during the anti-angiogenic therapies (54). The mechanisms that underline the chemotherapeutic–associated thrombosis is not fully understood. It appears that targeted therapies-mediated thromboembolism is associated with on-target effects. However, conventional chemotherapies-mediated thromboembolism attributed to off-target effects. Based on the preclinical and clinical studies, the proposed mechanisms include; (1) the activation or disruption of the endothelium, (2) decrease in anticoagulants and increase in procoagulants, such as TF (tissue factor), cytokine-controlled defective anticoagulant pathways, and changes in the fibrinolytic pathways, and (3) the activation of platelets (55).

#### VEGFis and TKIs

They impair the VEGF-mediated tissue-type plasminogen activator (t-PA) release (56), and elevate platelets and coagulation factors to induce thrombosis (57). Additionally, TKIs increase hematocrit and reduce NO- and PGI_2_-mediated anti-platelet activity (58). Accordingly, a meta-analysis in patients receiving TKIs demonstrated that the risk of myocardial infarction increased by 3.5-fold, and the development of arterial thrombosis by 1.8-fold, in the treated group (59). VEGFR inhibitors accelerate atherosclerosis and increase the risk of cholesterol embolization syndrome, leading to acute cardiovascular complications (60).

#### Anthracyclines

Doxorubicin has been shown to a significantly increase a risk of venous thrombosis by 16.0% (47). Several preclinical and clinical studies have showed that doxorubicin-mediated thrombogenic effects are resulted from an elevated prothrombotic state induced by (1) endothelial injury, (2) the down-regulation of the endothelium-based protein C anticoagulant pathway due to the reduced levels of endothelial protein C receptor in ECs, (3) an increased TF procoagulant activity, and (4) activated platelets (61). In patients with breast cancer, doxorubicin increases levels of thrombin-antithrombin complexes, protein C, and activated protein C (62). Its prothrombotic effects are also due to phosphatidylserine-bearing microparticle (MP) generation, promoting intracellular Ca^2+^ increase and ATP depletion in platelets (63). A dysfunction of the NADP-dependent mitochondrial enzyme aldehyde dehydrogenase-2 (ALDH2) in ECs is also involved in the development of doxorubicin-mediated vascular damage and thrombosis (64). Altered levels of endothelium-derived NRG-1, PGI_2_, and ET-1 from ECs can also contribute to anti-platelet activity of doxorubicin (65).

#### Alkylating Agents

Cyclophosphamide and its toxic metabolites stimulate activation and release of platelet factor 4 (PF-4) that initiates the cascade of thrombosis and the binding of oxidized low-density lipoprotein (LDL) to ECs, and aggravates monocyte adhesion to endothelium (41). Cyclophosphamide-induced intrapapillary micro emboli is prominent cause of the ischemic myocardial damage (41). It also fosters acute pericarditis, myocardial hemorrhage, and atrophic and focal necrosis with interstitial edema (66).

Cisplatin facilities endothelial damage, hypercoagulation measured by increased levels of thrombin-antithrombin complexes and D-dimer, and platelet aggregation *via* activation of the arachidonic acid pathway that forms several inflammatory and thrombogenic molecules (67). However, the absolute risk of venous thrombosis associated with this class of agent remains low.

#### Antimetabolites

5-FU damages ECs and provokes severe vessel leakage and subsequent thrombus formation (68, 69). Patients receiving a cisplatin-based regimen with epirubicin and 5-FU or capecitabine exhibited an incidence of venous thrombosis of 15.1% (70).

In general, anticancer agents have more pronounced effect of the incidence of venous thrombosis than arterial thrombosis. An anticoagulation therapy may be required.

## Anticancer Therapy-Mediated Impaired Vasculature and Drug Delivery

**Figure 3:**
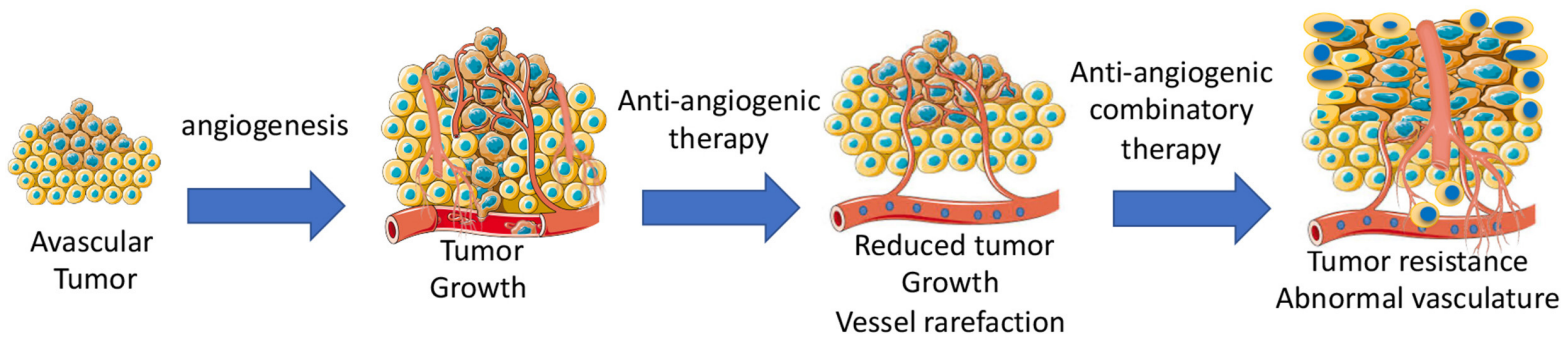
Development of angiogenesis and anti-angiogenic therapy-mediated development of tumor resistance due to abnormal tumor vasculatures.

Anti-angiogenic agents alone or in combination with other chemotherapeutics are widely used to inhibit tumor growth by targeting vascular network (71). Some types of cancers are sensitive to anti-angiogenic therapy, while other types of cancers are completely insensitive. Adaptation to microenvironment, such as metabolic changes (72) or autophagy (73), can determine whether a tumor is sensitive to anti-cancer treatments. Some tumors can initially respond, but then develop acquired resistance during the anti-angiogenic treatment due to activation of alternative pathways, such as vessel co-option and vessel mimicry (74). Development of hypoxia in tumors reduces the activity of the prolyl hydroxylase domain proteins (PHD1–3), and prevents the degradation of HIF-1α and HIF-2α (75). High levels of HIFs in turn increases the transcription of HIF-driven hypoxia-related genes, including the potent angiogenic factors, VEGF to form a neovascular network to further increase tumor growth. Indeed, long-term anti-angiogenic therapy promotes genetic instability in tumor ECs, and causes vascular permeability and metastasis (76). Additionally, tumor-associated macrophages can trans-differentiate into ECs (77). In this case, tumors become highly vascularized and also resistant to chemotherapies. Tumor cells including infiltrated immature myeloid cells (78), fibroblasts (79), and endothelial progenitor cells (80) integrate into vessels or release pro-angiogenic growth factors, such as prokineticin-2 (3) or PDGF-C (79), leading to worse outcomes of drug delivery, invasion, and metastasis (Figure 3).

## Detection of Endothelial Damage and Thrombosis

Endothelial damages alter the expression of adhesion molecules and increase levels of pro-inflammatory cytokines (81). Thus, expression of adhesion molecules such as E-selectin, endothelin-1, and vascular cell adhesion molecule-1 (VCAM-1) are biomarkers of endothelial damage (82). The elevated levels of pro-inflammatory cytokines such as C-reactive protein (CRP) and IL-6 are also indicators of EC damage (83). Because asymmetric dimethylarginine (ADMA) synthesized *via* arginine methylation inhibits eNOS and promotes superoxide generation (84), ADMA is a marker of ROS generation. Indeed, activated ECs initiates procoagulant activity by releasing endothelium-derived glycoproteins such as von Willebrand factor (vWF), NRG-1, soluble thrombomodulin (sTM), and tissue plasminogen factor (t-PA) (85). Thus, increase levels of vWF, NRG-1 sTM, and t-PA are also the indicators of procoagulant activity and thrombosis.

The detection of micro vessel architectural parameters by Magnetic Resonance Imaging (MRI), Vessel Architectural Imaging (VAI), Microvascular Density (MVD), Positron Emission Tomography (PET), 3D ultrasonography, and CT is necessary in the clinic to asses vascular damage and select a proper timing window for tumor vascular normalization by anti-angiogenic therapies (86).

## Drugs Protecting Endothelial Cell Damage Induced By Chemotherapeutic Agents

Angiotensin converting enzyme inhibitors (ACEi), NO donors, antioxidants, and statins have direct effects on ECs, while angiotensin receptor blockers (ARBs), renin inhibitors, beta blockers, and estrogens indirectly affect EC function. Beta blockers, thiazide diuretics, mineralocorticoid receptor antagonists are used as additional antihypertensive agents. Here we focus on the first group of the EC protective drugs.

### ACEis

ACEis ameliorates the left ventricular ejection fraction (LVEF) decline, when they are administered together or after anthracyclines. However, the vascular protective effects of ACEi, zofenoprilat, but not other ACEi (i.e., captopril or enalaprilat) are related with activation of survival pathways in cardiac cells, and its antioxidant and ROS scavenger properties. More specifically, zofenoprilat up-regulates the expression of eNOS, FGF-2, and telomerase (TERT) transcripts, thereby, promoting cell survival, rescuing damaged ECs, and inducing physiological angiogenesis without altering vascularization at tumors (87). Thus, zofenoprilat exerts its EC protective effects through off-target mechanisms, and may even maximize cytotoxic drug delivery to tumor cells (8).

### Nitric Oxide Donors, Antioxidants, and Statin

Novel NO donor drugs metal-nonoates (88) and the mitochondrial aldehyde dehydrogenase (ALDH2) activator, called Alda-1 may restore eNOS functioning, and FGF-2 production and release, thereby, protecting ECs against anticancer drug-mediated damages (89). ALDH2 plays a central role in the vasodilator actions of nitroglycerin, restores mitochondrial functions, and promotes vascular recovery of ischemic myocardium (90). However, high ALDH2 metabolic activities have been observed in tumor ECs as well. Thus, whether ALDH2 mitigates anti-cancer drug efficacy in tumor should be tested.

Many animal models showed that vitamin E, vitamin C, vitamin A, coenzyme Q, and flavonoids can reduce the anthracycline-mediated cardiovascular toxicity. However, clinical use of antioxidants to protect the heart during anthracycline chemotherapy is paved away due to reduce cytotoxic efficacy toward cancer cells (91).

All FDA-approved statins are effective in lowering serum cholesterol by inhibiting activity of 3-hydroxy-3-methyl-glutaryl-coenzyme A reductase (HMGCR), a rate-limiting enzyme of the mevalonate pathway, in the liver. Therefore, they are integrated into cancer patient care to protect against atherosclerosis development during anticancer therapies. However, epidemiologic studies demonstrated that statin type, dose, and treatment duration, statin sensitivity, and toxicity are all important variables to evaluate statins beneficial effects in adverse effects of anticancer drugs (92).

## New Horizons in Therapeutic Strategies: Pro-Angiogenic Therapy to Prevent Vascular Toxicity Without Altering Anti-Neoplastic Properties of Chemotherapeutics

### VEGF-B-Mediated Endothelial Protection Against Doxorubicin-Mediated Cardiotoxicity

Vascular endothelial growth factor-B (VEGF-B) promotes coronary arteriogenesis, physiological cardiac hypertrophy, and ischemia resistance. It also prevents doxorubicin-induced cardiotoxicity and congestive heart failure. A recent preclinical study has shown that pretreatment of tumor-bearing mice with an adeno-associated viral vector expressing VEGF-B completely inhibits the doxorubicin-induced cardiac atrophy and whole-body wasting (93). VEGF-B also alleviates capillary rarefaction in the heart and improves cardiac function in doxorubicin-treated mice. Indeed, VEGF-B protects EC from apoptosis and restores tube-formation capacity of ECs without altering anti-tumor role of doxorubicin. Importantly, VEGF-B does not affect serum or tissue concentrations of doxorubicin. By inhibiting doxorubicin-induced endothelial damage, VEGF-B could provide a novel therapeutic possibility for the prevention of chemotherapy-associated cardiotoxicity in cancer patients.

### Prokineticin Receptor-1 Signaling Inhibits Dose- and Time-Dependent Anthracycline-Induced Cardiovascular Toxicity *via* Myocardial and Vascular Protection

Prokineticins (PROK1 and PROK2) are neuropeptides/hormones that are mainly released by macrophages and reproduction organs in the peripheral system (94). They utilize two G-protein–coupled receptors (GPCRs) namely prokineticin receptors (PKR1 and PKR2). Expression of PROK2 and PKR1 levels are altered in patients with abdominal aortic rupture (8), during end-stage cardiac failure (95) after acute myocardial infarction (96), and in adipose tissues from obese patients (97). Interestingly, *PKR1* gene transfer improves survival and heart function in a mouse model of myocardial infarction (95) and promotes coronary arteriogenesis (98). However, *PKR2* overexpression in cardiomyocytes promotes pathological cardiac hypertrophy and causes vascular leakage (99, 100). These receptors have also divergent effects on ECs (101). Thus, a non-peptide agonist specific for PKR1, called IS20, was developed to mimic the cardioprotective effects of PROK2 against heart failure developed by myocardial infarction (102) and anthracyclines (6) in mice.

A recent preclinical study has demonstrated that prolonged exposure to low-dose doxorubicin does not induce apoptosis in ECs, but impairs angiogenesis (6). Importantly, IS20 restores doxorubicin-mediated cardiovascular toxicity by activating Akt or MAPK pathways. Genetic or pharmacological inactivation of PKR1 abolishes these effects of IS20. Mice exposed to chronic doxorubicin treatment exhibit apoptosis in cardiac cells, vascular rarefaction and fibrosis, consequently impaired systolic and diastolic cardiac function, and reduced survival rate. IS20 reverses these detrimental effects of doxorubicin. IS20 also does not alter the cytotoxicity or antitumor effects of doxorubicin in breast cancer lines or in a mouse model of breast cancer. Altogether, this study provides evidence that PKR-1 is a promising target to combat cardiovascular toxicity of cancer treatments (6).

## Conclusion and Perspectives

Anticancer treatments induce vascular damage, hypertension, and thrombosis, which affect survival and quality of life of the patient (Table 1). Therefore, pre-existing hypertension and a thrombosis risk assessment should be conducted before starting any type of chemotherapies (103). A continued characterization of changes of microvessel network patterns and blood pressure by anticancer drugs is necessary to prevent development of hypertension and organ damages, especially during the 1st cycle of therapy when the patients experience a secondary elevation in blood pressure.

**Table 1:** Vascular damages and diseases induced by the widely prescribed anticancer drugs.

**Anti-cancer drugs and their use in type of cancers**	**Vascular toxicity**	**Mechanism**	**Ref**.
**Anthracyclines (Doxorubicin)**Leukemia, lymphoma, melanoma, uterine, breast, and gastric cancers	* Vascular injury * Microvascular rarefaction * Endothelial dysfunction and hypertension * Hypercoagulation, thrombosis, & atherosclerosis	* Oxidative stress-mediated ROS accumulation * Apoptosis due to DNA interference * Disruption of the tight junction protein ZO-1 in ECs * Anti-angiogenesis * Mitochondrial DNA damage * The endothelium-based protein C anticoagulant pathway interruption	(18) (21) (22) (5) (49) (59)
**Tyrosine kinase inhibitors (TKI) and VEGF inhibitors (VEGFis)**Renal cell cancer, gastro-intestinal stromal tumors, hepatocellular cancer	* Microvascular rarefaction * Hypertension * Anti-platelet activity * Hypercoagulation & thrombosis, & atherosclerosis	* NRF2 downregulation * Anti-VEGF effect, ROS accumulation and disruption of NO levels and PI3K/Akt survival pathway. * Increased vasoconstriction due to disruption of both endothelium-dependent and -independent vasodilatation * Loss of pericytes due to inhibition of PDGF * Increased hematocrit and thrombogenesis activity by reducing NO- and PGI_2_-mediated anti-platelet activity * Atherosclerosis and increase in the risk of cholesterol embolization syndrome	(26) (28) (31) (40) (30) (56) (60)
**Cyclophosphamide**Breast cancer, lymphoid, and pediatric malignancies	* Vascular injury * Microvascular rarefaction * Hypercoagulation, thrombosis, & atherosclerosis	* Increased levels of the expression of tissue necrosis factor * Reduced levels of VEGFs * Activation of platelet factor 4 (PF-4)	(24) (33) (24)
**Cisplatin**Ovaries, testis cancers, solid tumors of the head and neck	* Vascular injury * Microvascular rarefaction * Endothelial dysfunction and hypertension * Hypercoagulation, thrombosis, & atherosclerosis	* Increased levels of EC- inflammatory substances to produce hydrogen peroxide * Increased platelet aggregation levels *via* the arachidonic acid pathway	(26) (34, 35) (51)
**5-Fluorouracil (5-FU)**Breast cancer, head and neck cancers, anal, stomach, colon cancers some skin cancers	* Vascular injury * Hypercoagulation, thrombosis, & atherosclerosis	* Ultrastructural changes in the endothelium of the heart * EC damage * Direct prothrombotic effect	(27) (65, 66)
**Taxanes**Breast, ovarian, lung, bladder, prostate, melanoma, esophageal, other types of solid tumor cancers	* Microvascular rarefaction	* Endothelial damage and impaired angiogenesis	(37)

Several mechanisms for anticancer drug-mediated vascular toxicity have been identified (104), however, there are still many unknown molecular processes that need to be unraveled to better understand exactly how anticancer treatments provoke vascular damages. Endothelial metabolism and new signaling pathways could be novel targets of the vascular protectant.

Identification of underlying pathological mechanisms of development of vascular toxicity is a key element to optimize benefits in tumor development and drug delivery of chemotherapies.

The improvement in cancer therapy of the past two decades is due to the development of numerous novel targeted therapies. These drugs are also used in combination with other new anti-cancer drugs including inhibitors of immune check points, poly (ADP-ribose) polymerase (PARP), and histone deacetylase (HDAC). However, most of these treatments also induce vascular toxicity, leading to hypertension, thromboembolism, vasculitis, development of atherosclerotic plaques, and fibrotic heart disease. More clinical trials of cancer therapies are needed to be better document the vascular complications of the chemotherapeutics.

Some of the new cardiovascular protectants including GPCR-targeted compounds are potential drug candidates to improve management and prevention of the cardio vascular toxicity of anti-cancer therapy (105). Whether, these potential vascular protective agents minimize thrombotic risk associated with chemotherapies should also be examined. Further, studies are also necessary to examine their effects on the efficacy of anti-tumor drugs.

## Author Contributions

AM, P-YH, and CGN: create the figure and contribute to the writing. LD, NB-J, and CGN: conribute writing. CGN: conribution to editing, designing, and orginization of the idea. All authors contributed to the article and approved the submitted version.

## Conflict of Interest

The authors declare that the research was conducted in the absence of any commercial or financial relationships that could be construed as a potential conflict of interest.

## Publisher's Note

All claims expressed in this article are solely those of the authors and do not necessarily represent those of their affiliated organizations, or those of the publisher, the editors and the reviewers. Any product that may be evaluated in this article, or claim that may be made by its manufacturer, is not guaranteed or endorsed by the publisher.
